# Gene flow in environmental *Legionella pneumophila* leads to genetic and pathogenic heterogeneity within a Legionnaires’ disease outbreak

**DOI:** 10.1186/s13059-014-0504-1

**Published:** 2014-11-03

**Authors:** Paul R McAdam, Charles W Vander Broek, Diane SJ Lindsay, Melissa J Ward, Mary F Hanson, Michael Gillies, Mick Watson, Joanne M Stevens, Giles F Edwards, J Ross Fitzgerald

**Affiliations:** The Roslin Institute and Edinburgh Infectious Diseases, University of Edinburgh, Easter Bush Campus, Midlothian, EH25 9RG United Kingdom; Scottish Haemophilus, Legionella, Meningococcus and Pneumococcus Reference Laboratory, NHS Greater Glasgow and Clyde, Glasgow Royal Infirmary, Glasgow, Glasgow G31 2ER United Kingdom; Centre for Immunity, Infection and Evolution, University of Edinburgh, Ashworth Laboratories, King’s Buildings, Edinburgh, EH9 3FL United Kingdom; Department of Laboratory Medicine, Royal Infirmary of Edinburgh, Edinburgh, EH16 4SA United Kingdom; Department of Critical Care Medicine, Royal Infirmary of Edinburgh, Edinburgh, EH16 4SA United Kingdom; Edinburgh Genomics, The Roslin Institute, University of Edinburgh, Easter Bush Campus, Midlothian, EH25 9RG United Kingdom

## Abstract

**Background:**

Legionnaires’ disease is a severe form of pneumonia caused by the environmental bacterium *Legionella pneumophila*. Outbreaks commonly affect people with known risk factors, but the genetic and pathogenic complexity of *L. pneumophila* within an outbreak is not well understood. Here, we investigate the etiology of the major Legionnaires’ disease outbreak that occurred in Edinburgh, UK, in 2012, by examining the evolutionary history, genome content, and virulence of *L. pneumophila* clinical isolates.

**Results:**

Our high resolution genomic approach reveals that the outbreak was caused by multiple genetic subtypes of *L. pneumophila*, the majority of which had diversified from a single progenitor through mutation, recombination, and horizontal gene transfer within an environmental reservoir prior to release. In addition, we discover that some patients were infected with multiple *L. pneumophila* subtypes, a finding which can affect the certainty of source attribution. Importantly, variation in the complement of type IV secretion systems encoded by different genetic subtypes correlates with virulence in a *Galleria mellonella* model of infection, revealing variation in pathogenic potential among the outbreak source population of *L. pneumophila*.

**Conclusions:**

Taken together, our study indicates previously cryptic levels of pathogen heterogeneity within a Legionnaires’ disease outbreak, a discovery that impacts on source attribution for future outbreak investigations. Furthermore, our data suggest that in addition to host immune status, pathogen diversity may be an important influence on the clinical outcome of individual outbreak infections.

**Electronic supplementary material:**

The online version of this article (doi:10.1186/s13059-014-0504-1) contains supplementary material, which is available to authorized users.

## Background

*Legionella pneumophila* is an ubiquitous intracellular pathogen of environmental protozoa, commonly found in freshwater reservoirs [1]. The bacterium can also cause human infections (legionellosis) and is a causative agent of the severe community-acquired pneumonia known as Legionnaires’ disease [2]. Infection may occur following exposure to contaminated aerosols, although some individuals can seroconvert without displaying any symptoms [3]. The risk of human exposure to aerosols containing *L. pneumophila* increases with elevated ambient temperature, vapor pressure, and bacterial density in the reservoir [4,5], while host factors such as male gender, age, smoking and underlying respiratory pathology are associated with increased risk of developing Legionnaires’ disease [6]. Studies to date have largely considered that each outbreak of legionellosis likely results from a point source of a clonal *L. pneumophila* population, although a recent study has reported multiple sequence types associated with a single patient [7].

The ability of *L. pneumophila* to establish infection within human host cells is mediated through type IV secretion systems (T4SSs), complexes of proteins homologous to conjugation systems that mediate the transfer of nucleoprotein complexes and proteins between cells [8,9]. Among *L. pneumophila* strains, three families of variably present T4SS have been identified; T4ASS, T4BSS and the genomic island-associated T4SS (GI-T4SS) [10-12]. T4ASS, encoded by the *L. pneumophila* Lvh locus, has a role in host-cell entry and intracellular replication [13], and contributes to establishment of infection at lower temperatures [14], while the Dot/Icm system encoded by a T4BSS is essential for intracellular replication [15-17]. The functions of many of the effector proteins are unclear but a large number demonstrate homology with eukaryotic proteins, which may allow *L. pneumophila* to modulate the host response through structural mimicry of host components [18-21].

The incidence of *L. pneumophila-*associated disease in Scotland, UK ranges from 15 to 40 cases per year, with approximately two-thirds of cases attributed to sporadic infections acquired during travel [22]. Between 31 May and 17 July 2012, a total of 56 confirmed and 36 suspected cases of Legionnaires’ disease were reported with an epidemiological link to the south-west region of Edinburgh in Scotland. Analysis of prevailing wind conditions preceding and during the outbreak suggested a cluster of water cooling towers in the northeast of the affected area as the likely source of aerosols containing *L. pneumophila* responsible for the outbreak [23]. Although the suspect cooling towers were extensively sampled for *Legionella* spp., cultures were not obtained from any of the environmental samples. Patients were linked to the outbreak based on the results of traditional typing methods for *L. pneumophila* of serogrouping, monoclonal antibody (mAb) subgrouping, and sequence-based typing (SBT) [23]. However, the low resolution of traditional typing methods limits their utility for investigating intra-clonal levels of diversity among strains from a single outbreak. A previous pilot study examined the potential application of whole genome sequencing (WGS) to Legionnaires’ disease outbreak investigation by sequencing seven *L. pneumophila* isolates from an outbreak in Hampshire, UK, and a possible source was inferred from the analysis which was consistent with previous assertions based on traditional epidemiological analysis [24]. In addition, a very recent WGS study of *L. pneumophila* isolates from Alcoy, Spain, highlighted an important role for recombination in the evolution of *L. pneumophila* populations leading to multiple genotypes within the same outbreak [25]. In the current study, WGS was applied to all 22 *L. pneumophila* clinical isolates cultured from the 2012 Edinburgh outbreak in order to investigate their genetic diversity, genome content and pathogenic potential. Unexpectedly, we discovered considerable genetic heterogeneity among the outbreak isolates which was the result of mutation, recombination and horizontal gene transfer within environmental populations prior to release. Multiple genetic subtypes were identified within individual patients, and strain-dependent differences in virulence were observed in a *Galleria mellonella* model of infection, consistent with variation in pathogenic potential among outbreak isolates. These data have important implications for source attribution in future Legionnaires’ disease outbreaks, and imply a putative role for *L. pneumophila* virulence determinants in the variable outcome of infections within an outbreak.

## Results and discussion

### Genomic epidemiology of the 2012 Edinburgh Legionnaires’ disease outbreak

Confirmed cases of Legionnaires’ disease were defined by clinical or radiological evidence of community-acquired pneumonia in conjunction with either isolation of *Legionella* species from respiratory secretions, detection of *L. pneumophila* antigen in urine, or a positive *L. pneumophila* serogroup (Sg) antibody response. Based on these criteria, 56 cases of Legionnaires’ disease were confirmed, while an additional 36 cases were classed as probable (based on a positive respiratory secretion *L. pneumophila* Sg 1 PCR), or suspected *L. pneumophila*. Isolates were cultured from 15 of 92 patients, a culture success rate consistent with previous studies, and linked to the outbreak on the basis of date and location of isolation. Results of typing techniques indicated all isolates to be *L. pneumophila* Sg 1, mAb subgroup Knoxville and sequence type (ST)191. Viable cultures were not obtained from any of the water samples from suspected outbreak sources precluding a definitive source attribution. In order to investigate the genetic relatedness of the clinical *L. pneumophila* isolates from the outbreak, we sequenced the genomes of all 22 isolates which included multiple colonies from the primary isolation plates of each of 4 patients (Table 1). An additional three contemporaneous clinical isolates that were epidemiologically unrelated to the Edinburgh outbreak, four environmental isolates of ST191 obtained previously in the UK, and a single Sg 1, ST591 isolate were sequenced to provide a phylogenetic context to the outbreak isolates (Table 1). Sequence assemblies resulted in 26 to 103 contigs per genome, with N50 values ranging from 81,559 to 718,197 bp (Table S1 in Additional file 1). Sequence analysis confirmed that 21 of the 22 sequenced isolates that were linked to the outbreak were ST191. However, isolate 12_4117 from patient 3, which had previously been reported to be Sg 1, ST191, was determined to be a novel sequence type ST1418. The original sample from patient 3 was re-examined and colonies of ST191 and ST148 isolates were identified from the same clinical sample, consistent with a co-infection of the patient with multiple sequence types of *L. pneumophila*.

**Table 1:** **Origin, traditional typing, and genomic subtyping of** ***L. pneumophila*** **outbreak isolates**

**Patient**	**Isolate**	**Date of isolation**	**Serogroup**	**mAb**	**ST**	**Genomic subtype**
**Edinburgh outbreak isolates**						
1	12_4030	31/05/2012	1	Knoxville	191	A
	12_4054	31/05/2012	1	Knoxville	191	C
2	12_4042	01/06/2012	1	Knoxville	191	A
3	12_4117	02/06/2012	10	NA	1418	NA
4	12_4058	03/06/2012	1	Knoxville	191	A
5	12_4053	04/06/2012	1	Knoxville	191	A
6	12_4561	06/06/2012	1	Knoxville	191	B
7	12_4169	06/06/2012	1	Knoxville	191	B
8	12_4555	06/06/2012	1	Knoxville	191	B
9	12_4563	07/06/2012	1	Knoxville	191	B
10	12_4499	08/06/2012	1	Knoxville	191	C
	12_4480	Not recorded	1	Knoxville	191	C
11	12_5064	08/06/2012	1	Knoxville	191	B
11	12_4437	12/06/2012	1	Knoxville	191	B
12	12_4240	11/06/2012	1	Knoxville	191	D
13	12_4903	17/06/2012	1	Knoxville	191	B
14	12_5223	20/06/2012	1	Knoxville	191	B
15	12_5251	29/06/2012	1	Knoxville	191	A
	12_5392	29/06/2012	1	Knoxville	191	A
	12_5383	29/06/2012	1	Knoxville	191	A
	12_5414	29/06/2012	1	Knoxville	191	A
	12_5415	29/06/2012	1	Knoxville	191	A
**Contemporary non-outbreak isolates**						
NA	12_3965	31/05/2012	1	Benidorm	42	NA
NA	12_4251	07/06/2012	1	Philadelphia	616	NA
NA	12_4904	21/06/2012	1	Philadelphia	37	NA
**Historical non-outbreak isolates**						
NA	H080160261	2008	6	NA	191	NA
NA	H080160262	2008	6	NA	191	NA
NA	H080160263	2008	6	NA	191	NA
NA	H064020049	2006	1	Allentown	591	NA
NA	H092620872	2009	6	NA	191	NA

mAb, monoclonal antibody subtype; NA, not applicable; ST, sequence type.

**Figure 1:**
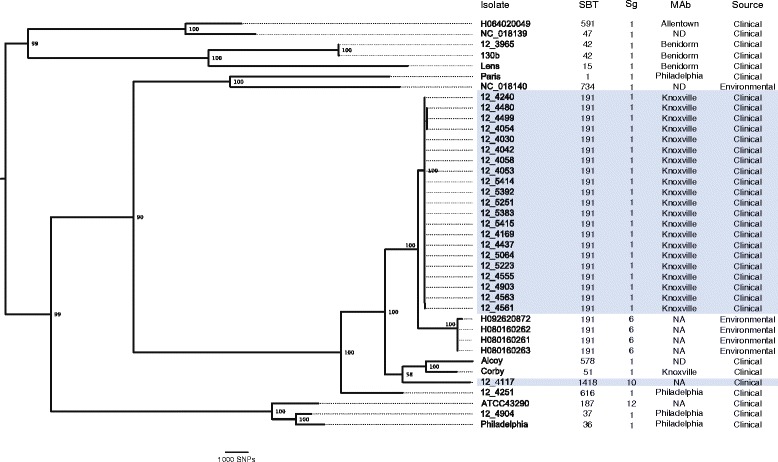
**The majority of Edinburgh Legionnaires’ disease outbreak isolates belong to a single ST191 clade.** Maximum-likelihood phylogeny based on the core genome of *L. pneumophila* outbreak and diverse reference isolates with *Legionella longbeachae* included as outgroup. Isolates from the Edinburgh Legionnaires’ disease outbreak are indicated in blue shading. Characteristics based on traditional typing schemes are denoted by: mAb, monoclonal antibody subgroup; NA, not applicable; ND, not determined; SBT, sequence-based typing; Sg, serogroup.

In order to investigate the relatedness of the outbreak isolates to the breadth of known *L. pneumophila* diversity, we reconstructed the phylogeny of all 30 sequenced isolates in addition to 9 additional *L. pneumophila* strains for which the genome sequence was publicly available (Table 2, Figure 1). The maximum-likelihood phylogeny indicated that the outbreak ST191 isolates were more closely related to each other than to non-outbreak isolates, but the non-outbreak, environmental ST191 isolates formed a sister clade in the phylogeny (Figure 1). The phylogeny also confirmed that the three *L. pneumophila* isolates obtained from cases of Legionnaires’ disease which were contemporaneous but had no epidemiological link to the Edinburgh outbreak (*L. pneumophila* strains 12_3965, 12_4251, and 12_4904) were not closely related to the outbreak isolates (Figure 1). Of note, while *L. pneumophila* Sg 1 has been responsible for the great majority of previously reported clinical infections [26], ST191 has not previously been reported as a common cause of legionellosis. However, it has been widely detected in environmental samples from the UK, Germany, the Netherlands, Poland, and Russia (PHE Legionella Database), highlighting its potential for future outbreaks. As stated, *Legionella* spp. were not cultured from any samples of the suspect water reservoirs implicated in the Edinburgh Legionnaires’ disease outbreak. In the future, culture-free sequencing techniques may be useful for identifying the existence of related genetic subtypes within suspected water sources or patient samples without the requirement for culture [27,28].

**Table 2:** **Origin and characteristics of previously sequenced** ***L. pneumophila*** **strains included in this study**

**Isolate**	**Isolation date**	**Isolation country**	**Sg**	**mAb**	**ST**	**Source**	**Reference**
Alcoy	1999	Spain	1	ND	578	Clinical	[10]
Corby	NA	UK	1	Knoxville	51	Clinical	[11]
ATCC43290	1987	USA	12	NA	187	Clinical	[29]
130b	1978	USA	1	Benidorm	42	Clinical	[30]
Lens	NA	France	1	Benidorm	15	Clinical	[19]
Paris	NA	France	1	Philadelphia	1	Clinical	[19]
Philadelphia	1974	USA	1	Philadelphia	36	Clinical	[31]
NC_018139	NA	France	1	ND	47	Clinical	[32]
NC_018140	NA	France	1	ND	734	Environmental	[32]

mAb, mAb, monoclonal antibody subtype; NA, not applicable; ND, not determined; Sg, serogroup; ST, sequence type.

### The Edinburgh Legionnaires’ disease outbreak was caused by multiple genetic subtypes of ST191 *L. pneumophila*

**Figure 2:**
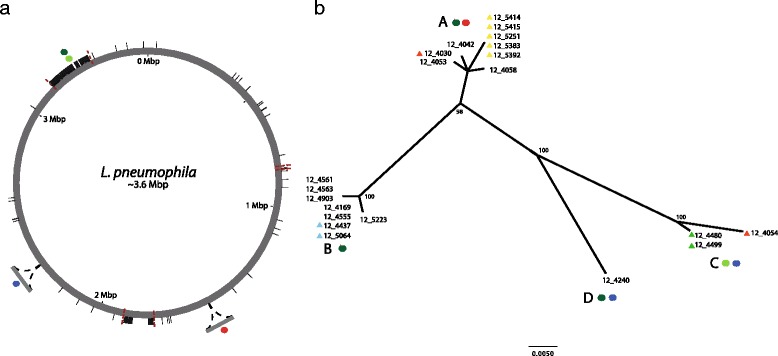
**ST191 outbreak isolates are represented by multiple genetic subtypes which arose by mutation, recombination and horizontal gene transfer. (a)** Genome distribution of mutations and predicted recombinant regions among ST191 outbreak isolates. Polymorphisms are mapped to the reference strain Corby. SNPs are represented by short black lines, and variant T4SSs are indicated by colored circles; Dot/Icm T4BSS (variant 1), dark green; Dot/Icm T4BSS (variant 2), light green; Lvh T4ASS (Philadelphia), red; Lvh T4ASS (novel), blue. Predicted recombinant regions are flanked by dashed red lines. **(b)** Maximum likelihood un-rooted radial phylogeny reconstructed using the non-recombinant core genome of ST191 outbreak isolates. For each node, maximum likelihood bootstrap values are displayed. Filled triangles indicate multiple isolates that were obtained from a single patient as follows; patient 1, yellow; patient 2, red; patient 12, green; patient 13, blue. Presence or absence of genomic regions encoding T4SS is indicated by colored filled circles as per **(a)**.

In order to elucidate the relatedness of the 21 ST191 outbreak isolates to each other, the core genome variation among the ST191 outbreak isolates only was examined. Inspection of the sequence alignment revealed the presence of three regions of high SNP density in each of three isolates (12_4480, 12_4499, 12_5054), which were suggestive of recombination events in those strains (Figure 2). Removal of these genomic regions yielded an alignment of 2,694,741 bp, with a total of 42 polymorphic sites (Figure 2). Phylogenetic reconstruction using maximum likelihood and Bayesian methods revealed four distinct subtypes (A to D) among the ST191 outbreak isolates from 15 patients (Figure 2, Table 1). Of note, for patients 10, 11, and 15 the multiple isolates obtained from each were identical, suggesting that the short incubation periods did not support extensive within-host diversification. However, isolates from patient 1 were represented by multiple genetic subtypes of ST191 (clades A and C), which were differentiated by 20 core genome SNPs (Figure 2)*.* Of the four subtypes, three were identified in multiple patients, including subtype A in four patients, subtype B in seven patients, and subtype C in two patients, indicating their wide distribution among patients infected during the outbreak (Table 1). The short timescale between exposure to and isolation of the pathogen during the outbreak and the lack of person-to-person transmission for *L. pneumophila* [33,34] strongly suggest that the genetic subtypes of ST191 existed in the outbreak source prior to release and evolved from a recent progenitor within the water reservoir by a combination of gene mutation and recombination.

As the ST191 isolate sequences in the current study did not facilitate determination of the mutation rate of the outbreak isolates (Figure S1 in Additional file 1), we estimated the lower limit of the time to the most recent common ancestor (tMRCA) of the ST191 outbreak isolates using a previous estimate of the evolutionary rate for *L. pneumophila* [25] as a prior in Bayesian phylogenetic analysis. This indicated that the MRCA likely existed many months prior to the outbreak (Table S2 in Additional file 1). Consistent with this, a previous study demonstrated that *L. pneumophila* can persist in cooling towers for periods of at least 5 years, a time-frame which could easily account for the identified genetic diversity among the ST191 isolates from the Edinburgh outbreak [35].

Finally, the identification of multiple *L. pneumophila* strains in patients 1 and 3 raises questions concerning our capacity to confidently link infections to a single environmental source in Legionnaires’ disease outbreak situations. Of note, a previous study demonstrated the presence of *L. pneumophila* with indistinguishable pulsed field gel electrophoresis profiles in multiple cooling towers within a 1 km radius, suggesting that cross-contamination of water cooling towers may lead to the existence of closely related subtypes in distinct reservoirs, thereby complicating source attribution [35].

### Outbreak strains differ in content of genes encoding T4SSs

Considering the existence of multiple genetic subtypes of the outbreak population of ST191 *L. pneumophila*, we also examined the variation in the accessory genome of ST191 isolates. Several regions of difference were identified among the isolates examined, including three genetic elements encoding T4SSs (Figure 2). For example, all nine isolates in clade A (Figure 2) contained a 39,441 bp Lvh T4ASS which shared 100% nucleotide identity with a genetic element in the genome of the Philadelphia 1 strain, derived from the original Legionnaire’s disease outbreak in Philadelphia in 1976 [31]. Of note, a recently sequenced genome of a Sg 6 isolate from Thunder Bay, Canada, contained the same genetic element with one SNP (among 39,441 bp) [36], demonstrating a remarkably high level of nucleotide conservation for the genetic element encoding this T4SS in clinical isolates obtained on two continents almost four decades apart. Isolates belonging to clades C and D had a novel 45 kb region not identified in the other outbreak isolates which contained 46 predicted coding sequences, including homologs of *lvrA*, *lvrB*, *lvrC*, and *virB4* (Table S3 in Additional file 1), suggesting a putative role as a novel Lvh T4ASS (Figure S2 in Additional file 1). In addition, all isolates contained a copy of the T4BSS encoding the *dot*/*icm* system, which has previously been demonstrated to have an essential role in pathogenesis, but a high density of polymorphic sites at the *dotA/icmVWX* locus differentiated the Dot/Icm T4BSS into 2 distinct molecular variants associated with 18 isolates in clades A, B, and D (variant 1), and 4 isolates in clade C (variant 2), respectively (Figure 2). Finally, although indistinguishable in the core genome, the two isolates from patient 13 of genetic subtype B differed by the presence a 55 kb element including genes encoding resistance to heavy metals, and a 2.7 kb region encoding two hypothetical proteins. In summary, the data indicate considerable variation in accessory genome content among isolates from a single outbreak. Taken together, our genome analysis revealed that *L. pneumophila* persisting within the outbreak source diversified through a combination of mutation and gene flow, including recombination and horizontal gene transfer, leading to a heterogeneous population responsible for the Edinburgh Legionnaires’ disease outbreak.

### Variation in T4SS gene content among outbreak isolates correlates with virulence in a *G. mellonella* infection model

**Figure 3:**
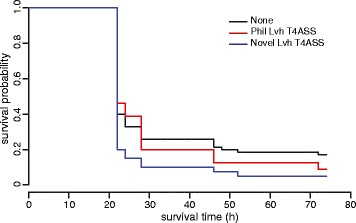
**The virulence of** ***L. pneumophila*** **outbreak isolates correlates with T4SS content.** *G. mellonella* survival curves representing the mean for isolates grouped according to the combination of T4SS, including presence of the Lvh T4ASS Philadelphia (red), Lvh T4ASS novel (blue), and isolates without T4ASS Philadelphia or T4ASS novel (black). Larvae infected with isolates which encoded the Lvh T4ASS novel had a lower survivability compared with those with infected other isolates (*P* = 0.04).

The T4SSs play a central role in the capacity of *L. pneumophila* to infect free-living amoeba and survive within vacuoles in human alveolar macrophages. Mouse models have traditionally been applied to examine the role of specific *Legionella* spp. determinants in pathogenesis but an infection model of the *G. mellonella* (waxmoth) larvae has been developed recently, providing an effective model of *L. pneumophila* human infection which allows analysis of T4SS-dependent virulence [37]. We used the *G. mellonella* infection model to examine the virulence of all *L. pneumophila* clinical isolates obtained in the 2012 Edinburgh outbreak, in addition to a reference strain *L. pneumophila* Paris of known virulence [37]. Considerable strain-dependent variation in *Galleria* host survival was observed after infection (Figure 3), and a significant difference in killing capacity was identified between groups of isolates with unique combinations of T4SSs. In particular, strains with the novel putative T4SS resulted in more rapid killing of *Galleria* larvae than strains without it (*P* = 0.04; Figure 3). There was no significant difference between isolates containing different variants of the Dot/Icm T4SS (data not shown). Analysis of clinical data for each patient for which there was comparable information (n = 13) was carried out. The small number of patients infected with the more virulent subtype containing the novel Lvh T4SS was not sufficiently powered to facilitate a robust statistical analysis, and there was no statistically significant difference in clinical disease indicators between patients infected with strains containing the novel Lvh T4SS (n = 3), and those infected with strains lacking the Lvh T4SS (n = 10). However, patients with the novel T4SS required more clinical care intervention, including higher intensive care unit (ICU) admission, a higher proportion requiring mechanical ventilation, and fewer ICU-free days. Taken together, we have identified heterogeneity in virulence among closely related *L. pneumophila* isolates from the same Legionnaires’ disease outbreak that may influence the outcome of infection.

## Conclusions

The application of genomics offers great potential for enhanced understanding of the biology of infectious disease outbreaks. Here, the high resolution of WGS revealed previously unappreciated levels of genetic and pathogenic complexity underlying a major Legionnaires’ disease outbreak. In particular, our data suggest that the genetic diversity of *L. pneumophila* environmental populations associated with an outbreak may make source attribution challenging, particularly in the light of potential cross-contamination of neighboring water coolers [35]. Intra- and inter-species horizontal gene transfer is common among *Legionella* spp., and variation in T4SS content between strains has been reported previously [10,13,30,32,38]. However, differences in T4SS content and associated virulence have not previously been described among strains from a single outbreak. While susceptibility to infection is strongly influenced by known host-associated risk factors, our data suggest that variation in content of virulence genes among outbreak isolates may also influence the clinical outcome of individual infections.

## Materials and methods

### *L. pneumophila* culture and DNA extraction

*L. pneumophila* was incubated on buffered charcoal yeast extract (BCYE) agar at 37°C for 48 h in a humid environment. A sweep of *L. pneumophila* was taken from the agar culture using a sterile loop, suspended in 2 ml phosphate-buffered saline (PBS), and pelleted by centrifugation for 10 minutes at 5,000 × g. Genomic DNA extraction was performed according to the standard isolation of DNA from Gram-negative bacteria protocol using the QIAcube platform (QIAGEN, Benelux B.V., Netherlands Netherlands).

### DNA sequencing, assembly and alignment

Genomic DNA libraries were prepared using the Illumina TruSeq kit, and sequenced using either 150 bp paired-end runs on an Illumina MiSeq, or 100 bp paired-end runs on an Illumina HiSeq 2000. A sequence project has been created at the European Nucleotide Archive with project accession PRJEB6631. The raw FASTQ files were examined for Illumina adaptor sequences using cutadapt v.1.2 [39] to facilitate removal of adaptor contamination. The 5' and 3' ends of reads were trimmed to remove low quality scoring bases (Q <30) using Sickle v.1.2 [40]. Reads containing low frequency sequencing errors were corrected with Quake v.0.3 using a k-mer size of 15 [41]. Processed sequence reads for each isolate were assembled *de novo* using the de Bruijn graph-based assembler Velvet v.1.1 [42]. Optimal k-mer length and coverage cutoff parameters were calculated using the VelvetOptimiser v.2.2.5 script [43]. Assembled contigs, along with the genome sequences of representative clinical and environmental isolates from public databases, were aligned using progressiveMauve with default parameters [44]. Locally collinear blocks not common to all sequences or <1,000 bp in length were removed, resulting in a gap-free core genome alignment.

### Mapping of Illumina sequence reads

Sequence reads were mapped to the genome sequence of *L. pneumophila* strain Corby (accession number NC_009494.2) using the Burrows-Wheeler Aligner v.0.6.2 [45] with the Smith-Waterman algorithm disabled. Base calls were made at sites that were covered by at least five sequencing reads, and a core genome alignment was produced, with the core genome defined as nucleotide positions with a base call in all isolates.

### Recombination detection

To assess the level of recombination among the sequences in the alignment, the gap-free genome alignments from progressiveMauve were used as input for BratNextGen [46]. One hundred iterations of recombination learning were performed, until parameters had converged.

### Phylogenetic reconstruction and dating analysis

Core genome alignments were used as input for maximum likelihood phylogenetic reconstruction in RAxML v.8 [47]). A GTR model of nucleotide substitution was applied with the gamma model of rate heterogeneity. Support for nodes was assessed using 1,000 bootstrap replicates, and species tree was rooted by using the sequence of a *Legionella longbeachae* isolate as outgroup. Core genome alignments of outbreak ST191 isolates were used as input for BEAST v.1.8.0 in order to estimate the date for the most recent common ancestor [48]. A HKY model of nucleotide substitution was applied with a gamma model of rate heterogeneity plus invariant sites. Three demographic models were investigated (constant, exponential, and Bayesian skyline), using an uncorrelated lognormal clock with the previously reported rate of evolution for *L. pneumophila* as a prior (1.39 × 10^-7^, 95% HPD intervals of 5.41 × 10^-8^ to 2.30 × 10^-7^) [25]. For each demographic model, 3 independent chains were run for 1 × 10^8^ generations, with sampling every 10,000 generations and 10% discarded as burn-in.

### Genome annotation and identification of variable gene content

Variable gene content among the outbreak isolates was examined using a combination of genome annotation and alignment. Predicted protein and RNA coding sequences in the assembled contigs for the sequenced isolates were annotated using the prokka pipeline v.1.5.2 [49], and a custom BLAST database of *Legionella* sequences. The Gram-negative option was specified to predict signal sequences appropriate for *L. pneumophila*. The annotated assembled contigs were aligned using Mugsy v.1r.2.2 [50] and gene presence or absence determined with mugsy-annotator v.0.5 [51].

### *G. mellonella* larvae infection model

Single colonies of *L. pneumophila* were incubated in ACES [*N*-(2-acetamido)-2-aminoethanesulfonic acid] yeast extract broth at 37°C for 21 h [30]. The OD_600_ of liquid cultures was adjusted to 0.5 using PBS. Groups of 10 *G. mellonella* larvae were injected with 10 μl of either PBS or 1.6 ± 0.4 × 10^7^ CFU bacteria. The larvae were incubated at 37°C for 74 h and checked periodically for death. Kaplan-Meier survival curves were produced using the Survival package for R v.3.0.2 [52]. Differences between survival probability distributions were assessed using a log-rank test.

### Patient clinical information

Clinical information on all patients infected in the outbreak was extracted from public health interviews and ‘travel diaries’ to ascertain place of residence and work, date of symptom onset and co-morbidities. Following the outbreak, data on all confirmed and probable cases were extracted from patients’ medical records. These data included age, gender, co-morbidities, hospital length of stay and hospital outcome. Documented cardiovascular disease (including a history of ischemic or valvular heart disease or heart failure) respiratory disease (asthma, chronic obstructive pulmonary disease or lung fibrosis), chronic kidney disease, chronic liver disease or immunosuppression (systemic steroids or immunosuppressant therapy) was recorded. Where patients were admitted to critical care, data on demographics, acute physiology, therapeutic interventions and outcome were extracted using the Scottish Intensive Care Society Audit Group’s data collection program *Wardwatcher*. CURB65 score was also calculated to assess severity of pneumonia at presentation. CURB65 is a clinical prediction score validated for predicting mortality in community-acquired pneumonia [53].

### Ethics and data permissions

This study was undertaken as part of the further analysis of the factors underlying an outbreak of Legionnaires’ disease [23] and under the auspices of the Incident Management Team. Advice was sought from the relevant research ethics committee and it was confirmed that these outbreak-related investigations did not require research ethics approval. All tissue samples were handled in line with the requirements of the Lothian Bioresource. The authors who reviewed the clinical and tissue data were the relevant members of the Incident Management Team and data were anonymized at the earliest opportunity to minimize the risk of disclosure. The NHS Lothian Caldicott Guardian oversaw the governance, advised on the data flows, reviewed the collected clinical data for disclosivity and approved the final manuscript.

### Data availability

The Illumina sequences generated and used in this study are deposited and available in the European Nucleotide Archive [54], along with the draft genome assembly contigs under project accession number PRJEB6631.

## Additional file

Additional file 1: Figure S1. Output from Path-O-Gen representing a regression plot of root-tip distances against isolation time for outbreak isolates indicating a lack of temporal signal in the outbreak isolate sequences. **Figure S2.** Schematic representation of the genomic region encoding a novel putative T4ASS in some ST191 isolates. Arrows represent predicted coding sequences (CDSs) identified using the prokka annotation pipeline, colored as follows: blue, functional homology to previously identified T4SS proteins; green, homology to integrase family proteins. Unlabeled CDSs encode predicted hypothetical proteins. **Table S1.** Sequence assembly metrics for each *L. pneumophila* isolate sequenced. **Table S2.** Estimated time to the most recent common ancestor for the outbreak ST191 isolates. **Table S3.** Nearest homologs for ORFs identified in the genomic island encoding the novel putative T4SS among Edinburgh outbreak *L. pneumophila*.

## Abbreviations

bp: base pair
ICU: intensive care unit
mAb: monoclonal antibody
PBS: phosphate-buffered saline
SBT: sequence-based typing
Sg: serogroup
SNP: single nucleotide polymorphism
ST: sequence type
T4SS: type IV secretion system
tMRCA: time to most recent common ancestor
WGS: whole genome sequencing
