# Supplementary material for: Gene flow in environmental Legionella pneumophila leads to genetic and pathogenic heterogeneity within a Legionnaires’ disease outbreak
Source: Genome Biol. 2014 Nov 3;15(11):504. doi: 10.1186/s13059-014-0504-1 (PMC4256819; doi:10.1186/s13059-014-0504-1)
Supplement: Additional file 1: Figure S1. — Output from Path-O-Gen representing a regression plot of root-tip distances against isolation time for outbreak isolates indicating a lack of temporal signal in the outbreak isolate sequences. Figure S2. Schematic representation of the genomic region encoding a novel putative T4ASS in some ST191 isolates. Arrows represent predicted coding sequences (CDSs) identified using the prokka annotation pipeline, colored as follows: blue, functional homology to previously identified T4SS proteins; green, homology to integrase family proteins. Unlabeled CDSs encode predicted hypothetical proteins. Table S1. Sequence assembly metrics for each L. pneumophila isolate sequenced. Table S2. Estimated time to the most recent common ancestor for the outbreak ST191 isolates. Table S3. Nearest homologs for ORFs identified in the genomic island encoding the novel putative T4SS among Edinburgh outbreak L. pneumophila. [file 13059_2014_504_MOESM1_ESM.pdf]

Additional file 1

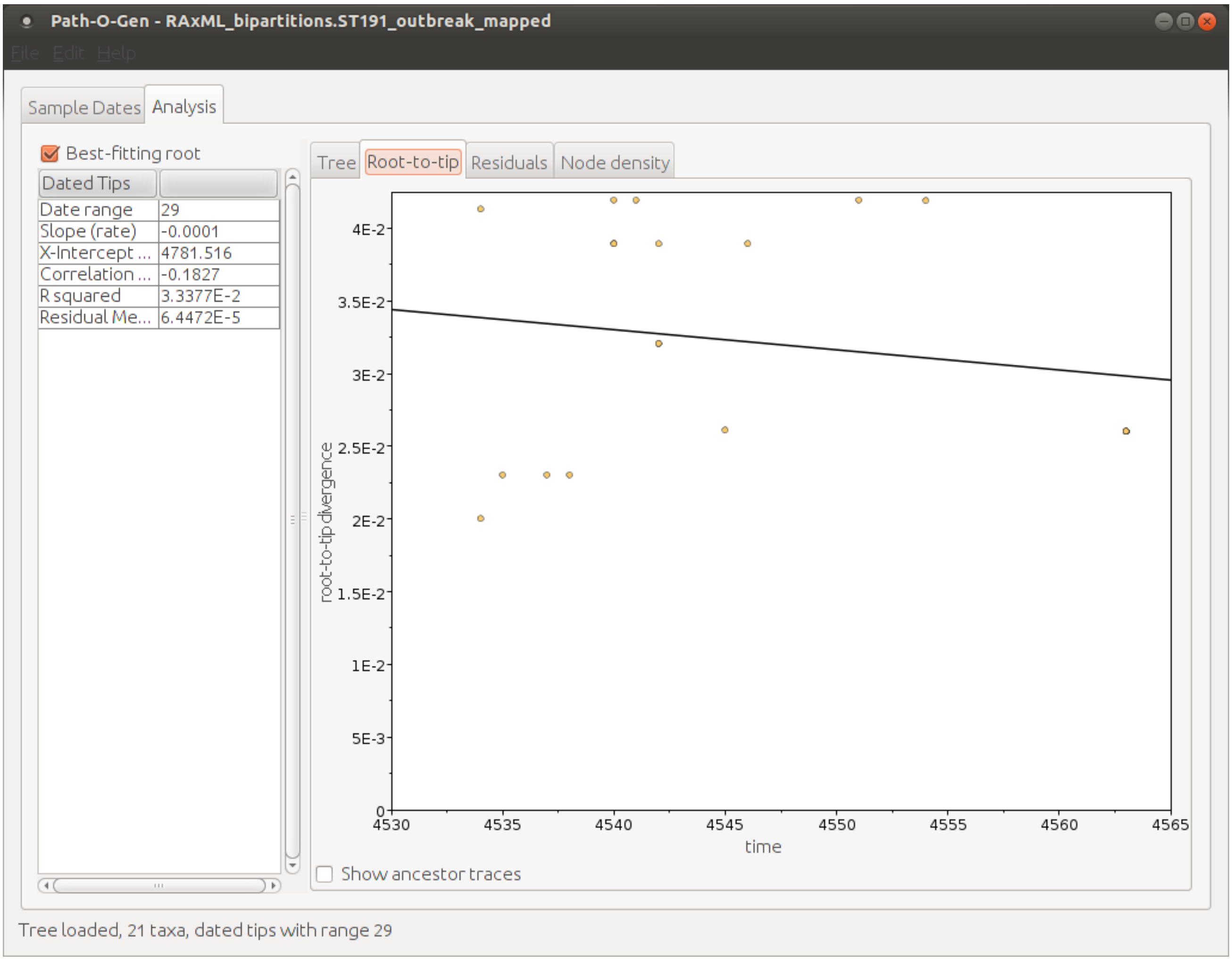

Fig. S1 Output from Path-O-Gen representing a regression plot of root-tip distances against isolation time for outbreak isolates indicating a lack of temporal signal in the outbreak isolate sequences.

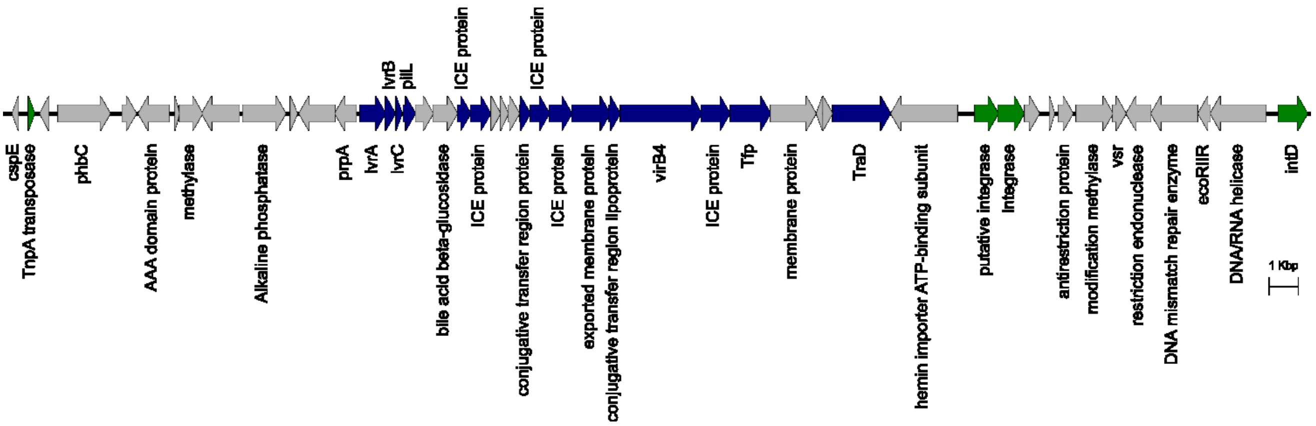

Fig. S2 Schematic diagram of the novel T4SS present in isolates from clades B, and D. Arrows represent predicted CDSs identified using the prokka annotation pipeline, coloured as follows: blue, functional homology to previously identified T4SS proteins; green, homology to integrase family proteins. Unlabeled CDSs are predicted hypothetical proteins.

Table S1. Sequence assembly metrics for each *L. pneumophila* isolate sequenced

Isolate	N50	Number of contigs	Accession number
12_4030	549491	29	ERS497042
12_4042	422611	32	ERS497043
12_4053	718197	34	ERS497044
12_4054	437914	32	ERS497045
12_4058	446387	35	ERS497046
12_4169	646293	26	ERS497048
12_4240	277640	32	ERS497049
12_4437	376046	31	ERS497051
12_4480	414859	36	ERS497052
12_4499	323143	36	ERS497053
12_4555	336849	27	ERS497054
12_4561	449291	33	ERS497055
12_4563	455502	32	ERS497056
12_4903	454289	32	ERS497057
12_5064	330767	27	ERS497059
12_5223	349034	28	ERS497060
12_5251	333356	29	ERS497061
12_5383	446507	35	ERS497063
12_5392	330608	29	ERS497062
12_5414	213979	38	ERS497064
12_5415	107106	74	ERS497065
12_3965	254145	42	ERS497041
12_4117	259870	51	ERS497047
12_4251	297133	47	ERS497050
12_4904	332618	46	ERS497058
H064020049	81559	103	ERS497066
H080160261	333314	28	ERS497067
H080160262	268362	37	ERS497068
H080160263	125881	82	ERS497069
H092620872	213979	39	ERS497070

Table S2. Estimated time of most recent common ancestor for the outbreak ST191 isolates. A HKY model of nucleotide substitution was applied with a gamma model of rate heterogeneity plus invariant sites. 3 demographic models were investigated (constant, exponential, and Bayesian skyline), using an uncorrelated lognormal clock with the previously reported rate of evolution for *L. pneumophila* as a prior (1.39×10^{-7}, 95% HPD intervals - $5.41 \times 10^{-8} – 2.30 \times 10^{-7}$) [1]. For each demographic model, 3 independent chains were run for 1×10^{8} generations, with sampling every 10,000 generations and 10% discarded as burn-in, resulting in uncertain but overlapping estimates for the time of the most recent common ancestor between 83 to 142 days prior to the start of the outbreak (median).

Demographic model	Mean root height (d)	Median root height (d)	95% HPDs
Constant	111	90	19-234
Exponential growth	94	83	22-192
Bayesian skyline	177	142	25-404

HPD, highest posterior density

Table S3 Nearest homologs for ORFs identified in the genomic island encoding the novel putative T4SS among Edinburgh outbreak *L. pneumophila*

ORF no.	Best BLASTp hit from nr database	evalue
ORF 1	ref|YP_003619374.1| hypothetical protein lpa_03002 [Legionella pneumophila 2300/99 Alcoy]	4e-27
ORF 2	ref|YP_003619373.1| hypothetical protein lpa_03001 [Legionella pneumophila 2300/99 Alcoy]	1e-67
ORF 3	ref|YP_001250835.1| polyhydroxyalkanoic synthase [Legionella pneumophila str. Corby]	0
ORF 4	ref|WP_019973975.1| hypothetical protein [Empedobacter brevis]	5e-24
ORF 5	ref|YP_001306333.1| putative transcriptional regulator [Thermosipho melanesiensis BI429]	5e-40
ORF 6	ref|WP_006870720.1| hypothetical protein [Legionella drancourtii]	2e-05
ORF 7	ref|WP_006870721.1| methylase [Legionella drancourtii]	3e-123
ORF 8	ref|WP_006870722.1| hypothetical protein [Legionella drancourtii]	0
ORF 9	ref|WP_006870723.1| transporter [Legionella drancourtii]	0
ORF 10	ref|WP_006870724.1| hypothetical protein [Legionella drancourtii]	4e-21
ORF 11	ref|NP_622719.1| hypothetical protein TTE1077 [Thermoanaerobacter tengcongensis MB4]	3e-93
ORF 12	ref|WP_006870727.1| repressor [Legionella drancourtii]	1e-102
ORF 13	ref|WP_006870728.1| hypothetical protein [Legionella drancourtii] (LvrA homolog)	1e-130
ORF 14	ref|WP_003632832.1| legionella vir region protein LvrB2 [Legionella longbeachae] (LvrB homolog)	3e-31
ORF 15	ref|YP_005186425.1| hypothetical protein lp12_2077 [Legionella pneumophila subsp. pneumophila ATCC 43290] (LvrC homolog)	7e-21
ORF 16	ref|WP_003632834.1| hypothetical protein [Legionella longbeachae] (PilL homolog)	6e-88
ORF 17	ref|WP_003632835.1| hypothetical protein [Legionella longbeachae]	9e-128
ORF 18	ref|WP_003632836.1| hypothetical protein [Legionella longbeachae]	0
ORF 19	ref|WP_003632837.1| hypothetical protein [Legionella longbeachae]	2e-97
ORF 20	ref|WP_003632838.1| hypothetical protein [Legionella longbeachae]	2e-109
ORF 21	ref|WP_006870735.1| hypothetical protein [Legionella drancourtii]	2e-57
ORF 22	ref|WP_003632841.1| hypothetical protein [Legionella longbeachae]	2e-51
ORF 23	ref|WP_003632842.1| hypothetical protein [Legionella longbeachae]	3e-85
ORF 24	ref|WP_003632843.1| hypothetical protein [Legionella longbeachae]	6e-79
ORF 25	ref|WP_003632844.1| hypothetical protein [Legionella longbeachae]	3e-147
ORF 26	ref|WP_003632845.1| hypothetical protein [Legionella longbeachae]	0
ORF 27	ref|WP_003632846.1| membrane protein [Legionella longbeachae]	0
ORF 28	ref|WP_003632847.1| hypothetical protein [Legionella longbeachae]	2e-89
ORF 29	ref|WP_003632848.1| type IV secretory protein VirB4 [Legionella longbeachae]	0
ORF 30	ref|WP_003632849.1| hypothetical protein [Legionella longbeachae]	0

ORF no.	Best BLASTp hit from nr database	evalue
ORF 31	ref|WP_003632850.1| pilus assembly protein PilT [Legionella longbeachae]	0
ORF 32	ref|WP_003632852.1| membrane protein [Legionella longbeachae]	0
ORF 33	ref|WP_003632853.1| hypothetical protein [Legionella longbeachae]	1e-38
ORF 34	ref|WP_003632854.1| hypothetical protein [Legionella longbeachae]	4e-45
ORF 35	ref|WP_003632855.1| conjugal transfer protein TraD [Legionella longbeachae]	0
ORF 36	ref|YP_007652706.1| Hypothetical protein XCAW_a00004 [Xanthomonas citri subsp. citri Aw12879]	2e-61
ORF 37	ref|WP_003632859.1| integrase [Legionella longbeachae]	0
ORF 38	ref|WP_003632860.1| integrase [Legionella longbeachae]	0
ORF 39	ref|WP_019646951.1| capsid protein [Novispirillum itersonii]	0.1
ORF 40	ref|WP_006869764.1| hypothetical protein [Legionella drancourtii]	5e-07
ORF 41	ref|WP_006869765.1| antirestriction protein [Legionella drancourtii]	7e-90
ORF 42	ref|YP_910577.1| DNA-cytosine methyltransferase [Chlorobium phaeobacteroides DSM 266]	0
ORF 43	ref|WP_017275581.1| DNA glycosylase [Sinorhizobium meliloti]	3e-56
ORF 44	ref|YP_001950539.1| HNH endonuclease [Geobacter lovleyi SZ]	8e-77
ORF 45	ref|YP_001950540.1| hypothetical protein Glov_0291 [Geobacter lovleyi SZ]	0
ORF 46	ref|YP_001950541.1| hypothetical protein Glov_0292 [Geobacter lovleyi SZ]	5e-49
ORF 47	ref|YP_001950542.1| non-specific serine/threonine protein kinase [Geobacter lovleyi SZ]	0
ORF 48	ref|YP_096070.1| prophage DLP12 integrase [Legionella pneumophila subsp. pneumophila str. Philadelphia 1]	1e-170

1. Sanchez-Buso L, Comas I, Jorques G, Gonzalez-Candelas F: **Recombination drives genome evolution in outbreak-related Legionella pneumophila isolates.** *Nat Genet* 2014, **advance online publication**.
